# Quantitative Assessment of Postoperative Lumbar Morphology Using Geometric Morphometrics: Within-Subject Changes Following TLIF and Decompression

**DOI:** 10.3390/diagnostics16193177

**Published:** 2026-09-30

**Authors:** Amit Benady, Owen Zhou, Amit Hadad, Hila May, Ryan David Quarrington, Afek Ash, Stone Sima, Victor Chen, Morsi Khashan, Ashish Diwan

**Affiliations:** 1 Spine Labs, St. George and Sutherland Clinical School, University of New South Wales, Sydney, NSW 2217, Australia; o.zhou@student.unsw.edu.au (O.Z.); s.sima@student.unsw.edu.au (S.S.); victor.chen@student.unsw.edu.au (V.C.); ashish.diwan@adelaide.edu.au (A.D.); 2 Department of Orthopedic Surgery, Tel Aviv Medical Center, Affiliated with Tel Aviv University, Tel Aviv 6997801, Israel; morsi.kh@gmail.com; 3 Levin Center for Surgical Innovation, Tel Aviv Medical Center, Affiliated with Tel Aviv University, Tel Aviv 6997801, Israel; afekash31@gmail.com; 4 Department of Anatomy and Anthropology, Gray Faculty of Medical & Health Sciences, Tel Aviv University, Tel Aviv 6997801, Israel; amthdd@gmail.com (A.H.); mayhila@tauex.tau.ac.il (H.M.); 5 Dan David Center for Human Evolution and Bio-History Research, Gray Faculty of Medical & Health Sciences, Tel Aviv University, Tel Aviv 6997801, Israel; 6 Department of Physical Education, Faculty of Sciences, Kibbutzim College of Education, Technology and the Arts, Tel Aviv 6250769, Israel; 7 Centre for Orthopedics and Trauma Research, Adelaide Medical School, The University of Adelaide, Adelaide, SA 5005, Australia; ryan.quarrington@adelaide.edu.au; 8 Department of Neurosurgery, Spine Unit, Tel Aviv Medical Center, Affiliated with Tel Aviv University, Tel Aviv 6997801, Israel; 9 Spine Service, Department of Orthopedic Surgery, St. George and Sutherland Clinical School, University of New South Wales, Sydney, NSW 2217, Australia; 10 Spinal Unit, Discipline of Orthopedic Surgery, Royal Adelaide Hospital, Adelaide, SA 5000, Australia

**Keywords:** geometric morphometrics, Procrustes analysis, TLIF, decompression, Cobb angle, sagittal alignment, lumbar lordosis

## Abstract

**Background:** Conventional radiographic assessment of postoperative lumbar alignment relies predominantly on Cobb angle measurements, which incompletely characterize global spinal morphology. We evaluated whether geometric morphometrics (GM) provides complementary quantitative information for assessing postoperative lumbar morphology after TLIF and decompression. **Methods:** Ninety-nine patients (TLIF *n* = 70; decompression *n* = 29) underwent landmark-based GM analysis of pre- and post-surgical lateral lumbar radiographs (48 landmarks, L1–S1). Because TLIF and decompression patients were imaged at markedly different postoperative intervals as a matter of routine clinical practice, the primary analyses tested whether each technique’s own pre-to-post shape change, adjusted for baseline L1–S1 angle, log centroid size, sex, age, number of operated levels, and degenerative spondylolisthesis, was significant (sign-flip permutation test), along with lordosis change (paired *t*-test, unadjusted). Cobb analyses assessed global lumbar lordosis (L1–S1) and operated level(s). **Results:** TLIF showed a significant within-group reduction in global lumbar lordosis at early postoperative imaging (Δ = −3.75°, paired *t*-test *p* < 0.001) and a significant, systematic change in overall lumbar shape (sign-flip permutation *p* < 0.001); decompression showed no significant within-group change in global lordosis (Δ = +0.47°, *p* = 0.733) or overall shape (*p* = 0.31), but a small increase in segmental lordosis at the operated level(s) (Δ = +1.44°, *p* = 0.036) that was not robust to sensitivity analysis. **Conclusions:** In this retrospective cohort, TLIF was associated with reduced lumbar lordosis and a systematic shape change at early postoperative imaging, while decompression showed preserved global lordosis and a small gain in segmental lordosis at long-term imaging. GM provided a consistent quantitative framework, with strong correlation and level-dependent agreement against manual Cobb measurement, for characterizing these within-technique postoperative changes.

## 1. Introduction

Degenerative lumbar spine pathology is a leading cause of low back pain, disability, and lost productivity in adults and underlies a major part of elective lumbar surgical procedures performed worldwide [1,2]. Posterior decompression, with or without instrumented fusion, remains the mainstay of surgical treatment for symptomatic lumbar spinal stenosis and degenerative spondylolisthesis [3,4,5]. When fusion is performed, transforaminal lumbar interbody fusion (TLIF) has emerged as one of the most widely adopted techniques owing to its unilateral posterior approach and its capacity for direct disc-space restoration through an interbody cage [6,7]. However, the choice between fusion-augmented and decompression-alone surgery remains a topic of active debate [5,8,9].

Sagittal alignment, and lumbar lordosis in particular, is recognized as a critical determinant of clinical outcome following lumbar surgery [10,11]. Loss of lumbar lordosis has been associated with worse patient-reported outcomes [12], persistent low back pain, and an elevated risk of adjacent segment degeneration [13,14]. TLIF is hypothesized to preserve or restore segmental lordosis through the structural support provided by the hardware, whereas decompression alone, particularly when it includes partial or total facetectomy, may alter sagittal alignment through disruption of the posterior tension band [15,16,17,18]. Evidence on the magnitude and pattern of post-operative morphological change following each technique remains limited.

Conventional radiographic assessment of lumbar morphology relies on Cobb angle measurement, a one-dimensional summary of vertebral endplate tilt that does not characterize the broader configuration of the lumbar spine [19]. Geometric morphometrics (GM) addresses this limitation by jointly modelling all anatomical landmarks and isolating shape variation from confounding effects of size, position, and orientation [20,21,22,23,24], but it has not been used specifically to characterize postoperative shape change following lumbar decompression or fusion surgery, which is the gap this study addresses. As such, GM has the potential to provide complementary quantitative information beyond conventional radiographic measurements while remaining directly relatable to established clinical metrics. The combination of GM with traditional Cobb angle measurement allows for simultaneous characterization of overall lumbar morphology and specific clinically meaningful angular dimensions.

This study aimed to characterize the within-subject postoperative change in lumbar shape and lordosis following posterior decompression and TLIF, using complementary GM and Cobb angle analyses. We hypothesized that TLIF would preserve or restore segmental lordosis through the structural support of interbody instrumentation [15,17] and that decompression by foraminolaminotomy, a limited procedure that preserves the midline posterior tension band, would not significantly alter lumbar lordosis [16,18]. The whole-shape GM analysis was exploratory and descriptive, undertaken to characterize the pattern of shape change accompanying these hypothesized lordosis changes; only the lordosis-specific predictions above were formally hypothesized a priori.

## 2. Methods

### 2.1. Study Design and Participants

This retrospective cohort study was conducted in accordance with the Declaration of Helsinki and was approved by the University of New South Wales Human Research Ethics Committee (Approval No. HC210096). Informed consent was waived due to the retrospective nature of the study. Pre- and post-surgical lateral lumbar spine radiographs from patients who underwent elective lumbar surgery at a single center between January 2020 and June 2024 were analyzed. The cohort comprised 99 patients (TLIF, *n* = 70; posterior decompression without fusion (foraminolaminotomy), *n* = 29). Each patient contributed one pre-surgical and one post-surgical radiograph (198 images in total). Mean age was 58.6 ± 13.3 years in the TLIF group (43 [61.4%] females) and 60.8 ± 14.5 years in the decompression group (10 [34.5%] females). The interval between surgery and the postoperative radiograph was recorded for each patient. This interval was 2 days (IQR 1–52) for the TLIF group and 329 days (IQR 92–588) for the decompression group. Inclusion criteria were (i) elective single- or multi-level lumbar surgery at L1–S1, (ii) availability of both pre-surgical and post-surgical standing lateral lumbar radiographs, and (iii) availability of complete clinical covariate data. Exclusion criteria were (i) pre- or post-surgical radiographs of insufficient quality for reliable landmark placement (e.g., inadequate field of view, excessive rotation, or motion artifact) and (ii) lumbar pathology of non-degenerative etiology (e.g., fracture, tumor, or infection). Of the 204 patients who underwent lumbar surgery at this center during the study period, 104 were excluded (76 lacking a postoperative radiograph, 20 lacking a preoperative radiograph, and 8 excluded per the criteria above despite having both images), leaving 100 who met inclusion criteria; one further patient was excluded during analysis after data review due to insufficient radiographic quality for reliable landmark placement identified on closer inspection, yielding the final cohort of 99. For each patient, age, sex, presence of degenerative spondylolisthesis, number of surgical levels, and Pfirrmann grade of the most degenerated disc were collected.

### 2.2. Landmark Placement

**Figure 1:**
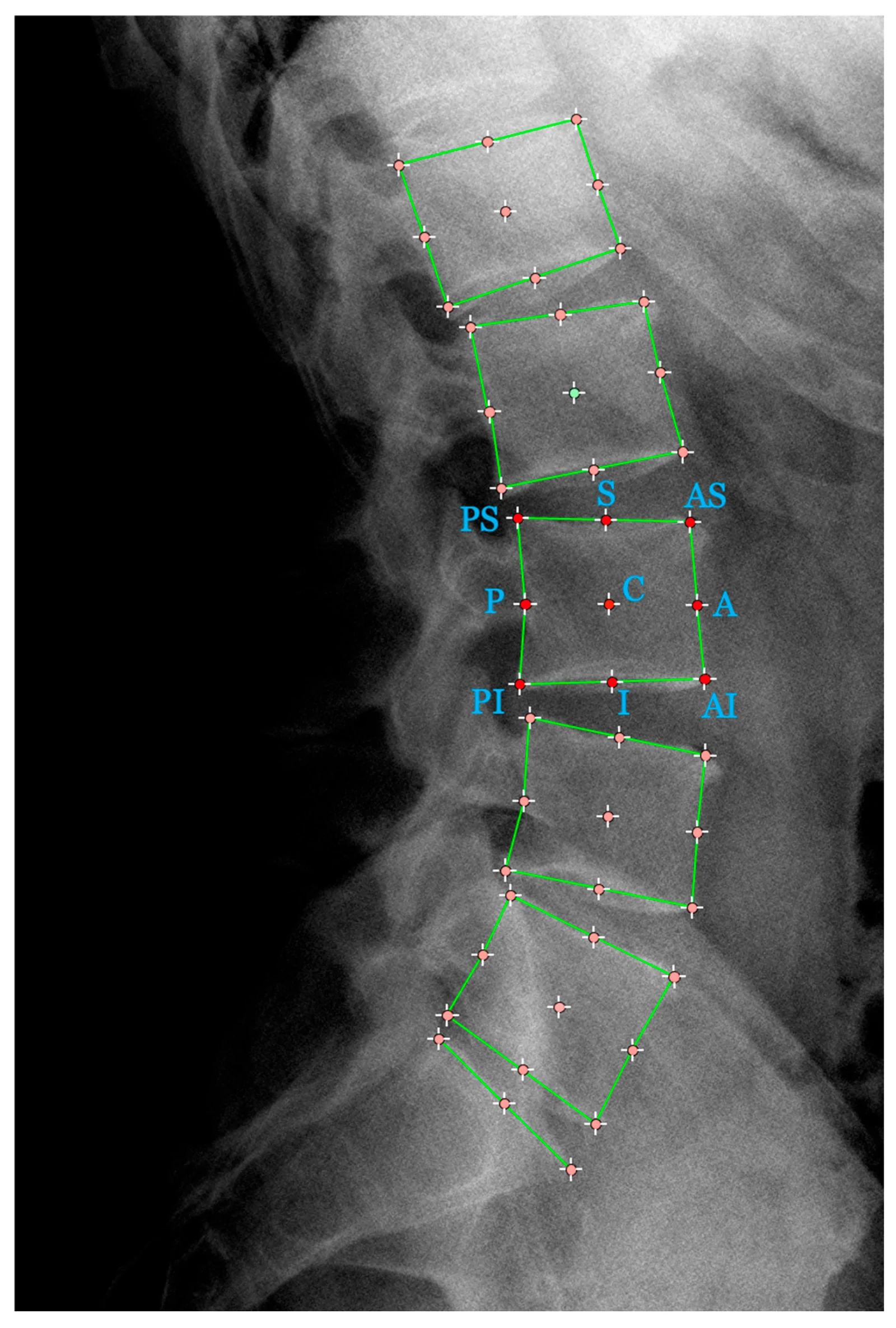
Landmark placement on lumbar vertebral bodies. Nine landmarks were placed on each vertebra (L1–L5) and three on the superior endplate of S1, yielding a total of 48 landmarks. A wireframe outline connects the landmarks for each vertebra. Landmarks include antero-superior (AS), antero-inferior (AI), postero-superior (PS), postero-inferior (PI), anterior wall midpoint (A), posterior wall midpoint (P), superior endplate midpoint (S), inferior endplate midpoint (I), and center of vertebral body (C). For S1, only three landmarks were placed on the superior endplate (AS, S, and PS).

All landmark coordinates were digitized on standing lateral lumbar radiographs using a custom script for Fiji-ImageJ software (v1.54i, National Institutes of Health, USA) by a single trained observer. A nine-landmark configuration was applied to each lumbar vertebra (Figure 1), comprising antero-superior (AS), antero-inferior (AI), postero-superior (PS), postero-inferior (PI), anterior wall midpoint (A), posterior wall midpoint (P), superior endplate midpoint (S), inferior endplate midpoint (I), and center of vertebral body (C). For S1, only three landmarks were placed on the superior endplate (AS, S, and PS). Corner landmarks (AS, PS, AI, and PI) were placed manually; midpoints (S, I, A, and P) were calculated automatically as endplate midpoints; wall midpoints were adjustable along the x-axis to the vertebral cortex, and C was derived as the centroid of S, A, I, and P. To assess landmark placement reliability, 10 patients (20 images: one pre- and one postoperative radiograph each) were randomly selected and independently digitized by two observers, who were blinded to the surgical group during digitization; complete blinding could not be maintained for TLIF postoperative images, in which interbody and pedicle-screw instrumentation is directly visible. The digitization procedure was repeated after a two-week interval. Inter-rater reliability was assessed between observers, and intra-rater reliability was assessed by comparing repeated measurements from the same observer. Reliability was evaluated using the intraclass correlation coefficient (ICC) under a two-way random-effects model for absolute agreement with single measures [ICC (2,1)].

### 2.3. Geometric Morphometric Analysis

A single full-lumbar landmark configuration was analyzed, comprising all 48 landmarks spanning L1 through S1. Generalized Procrustes Analysis (GPA) was performed, removing differences in position, scale, and orientation while preserving shape. Procrustes distance from the mean configuration was computed for each image; no image exceeded the 99th-percentile threshold for outlier exclusion, and all 198 images were retained for downstream analysis.

The covariate-adjusted residual model was then fitted with the retained covariates only (i.e., excluding group, timepoint, and their interaction; see Supplementary Tables S2 and S3 for full details). Procrustes residuals were computed by subtracting the fitted covariate effects from the GPA-aligned coordinates. Covariate-adjusted principal component analysis (PCA) was performed on these residual shapes for visualization of the morphospace; the resulting PC scores represent shape variation independent of the retained covariates.

### 2.4. Cobb Angle Computation and Analysis

For each image, Cobb angles were computed directly from the landmark coordinates using the standard outer-endplate convention: the angle subtended by the upper vertebra’s superior endplate (defined by the antero-superior and postero-superior corner landmarks) and the lower vertebra’s inferior endplate (defined by the antero-inferior and postero-inferior corner landmarks). Cobb angles were computed per image: segmental L1–2, L2–L3, L3–L4 and L4–L5; regional L1–L5; and global L1–S1 (computed against the sacral plateau, defined by the S1 antero-superior and postero-superior landmarks). Segmental Cobb angles for L1–L2 through L4–L5 were computed directly from each vertebra’s superior and inferior corner landmarks. Because only the superior (sacral plateau) landmarks were placed on S1, an L5–S1 segmental angle could not be computed the same way; it was instead derived as the difference between the global L1–S1 angle and the regional L1–L5 angle, algebraically equivalent to the angle between the L5 inferior endplate and the S1 superior endplate. This derived L5–S1 angle was included, where relevant, in the operated-level analysis. Lordosis was coded as positive and kyphosis as negative.

### 2.5. Statistical Analysis

Global lumbar shape was analyzed by Procrustes ANOVA using residual randomization in a permutation procedure (RRPP, 9999 permutations) with Type II sums of squares. Candidate covariates included baseline (pre-surgical) L1–S1 angle, log centroid size, sex, age, number of operated levels (Supplementary Table S1), degenerative spondylolisthesis, and Pfirrmann grade. These candidates were selected a priori on clinical grounds as plausible confounders of postoperative lumbar shape and lordosis; statistical significance in the screening model described below was used only as a secondary filter for retention in the final adjusted model, not as the basis for candidate selection itself. The full model also included the surgical group (TLIF vs. decompression) and the timepoint (pre vs. post) and their interaction and was used only to screen candidate covariates for retention; covariates that did not reach statistical significance (α = 0.05) in the full model were dropped from the retained set used in the residual model. Because this screening model pooled pre- and postoperative images without modelling within-patient dependence, its Group, Timepoint, and Group × Timepoint terms were not used for inference. The primary within-technique shape-change analyses were instead based on repeated-measures-safe within-patient change vectors, as described below.

Within-group pre- to post-surgical changes in Cobb-derived lordosis were summarized as mean Δ (post − pre) ± SD and tested with paired *t*-tests for global lumbar lordosis (L1–S1) and for the operated spinal level(s), the latter defined, for each patient, as the mean change in segmental Cobb angle across the level(s) identified from the surgical record. For multilevel procedures, averaging provided a single patient-level summary of the treated region and prevented patients with more operated levels from contributing disproportionately to the analysis.

Landmark-derived Cobb angles were compared with independent manual Cobb measurements performed by a single blinded observer across all 198 images. Within-patient shape change was quantified for each patient as the Procrustes distance between the pre- and post-operative configurations; the presence of a systematic (directional) within-group change was tested by a sign-flip permutation test (9999 permutations) of the mean shape-change vector. To relate shape to lordosis, the first principal component of the GPA-aligned coordinates was descriptively correlated with the L1–S1 Cobb angle across all images (not accounting for within-patient clustering), and the within-patient change in this component was correlated, as an independent-observations statistic, with the change in L1–S1 lordosis.

After assessing normality with the Shapiro–Wilk test, continuous variables were compared between groups with independent-samples *t*-tests (age, baseline L1–S1 angle), ordinal variables with the Mann–Whitney U test (Pfirrmann grade, number of operated levels), and categorical variables with Pearson χ^2^ tests (sex, degenerative spondylolisthesis). Agreement between GM-derived and manual Cobb angles was assessed by Pearson correlation and Bland–Altman analysis. The within-group paired *t*-tests were evaluated at the standard two-sided α = 0.05, as each of the four within-group comparisons (TLIF and decompression, at global and operated levels) is a distinct, pre-specified, hypothesis-driven test for its own technique rather than a family of comparisons addressing the same question and was therefore not subject to multiple-testing correction, unlike the two pre-specified between-group Cobb models, which were tested as a family against a Bonferroni-corrected threshold (Supplementary Methods). Covariate and landmark data were complete for all patients. All analyses were done using R version 4.5.2, with the geomorph 4.0.10, RRPP 2.1.2, car 3.1.5, readxl 1.4.5, lme4 2.0.1, and pwr 1.3.0 packages.

## 3. Results

### 3.1. Patient Cohort and Baseline Characteristics

Ninety-nine patients undergoing lumbar surgery were included. Of those, 70 underwent TLIF, and 29 underwent decompression alone (Table 1). The two groups did not differ significantly for age (*p* = 0.485), Pfirrmann grade (*p* = 0.826), prevalence of degenerative spondylolisthesis (*p* = 0.180), number of operated levels (*p* = 0.079), and pelvic incidence (60.3° ± 12.7° TLIF vs. 57.9° ± 9.3° decompression, available for 97/99 patients; *p* = 0.31). Sex distribution differed between the groups: in the TLIF group, females were more prevalent than males (61.4% vs. 38.6%), whereas in the decompression group, males were more prevalent than females (65.5% vs. 34.5%; Pearson χ^2^ test, *p* = 0.026). Decompression patients had lower baseline lumbar lordosis at the landmark-derived L1–S1 angle (47.33° ± 13.77° vs. 53.42° ± 10.40° for TLIF; *p* = 0.038). Baseline L1–S1 lordosis was modestly greater in women than in men (53.9° vs. 49.0°; *p* = 0.04); however, the lower baseline lordosis of the decompression group was similar in magnitude within each sex (women: decompression 48.0° vs. TLIF 55.3°; men: decompression 47.0° vs. TLIF 50.5°), and a formal adjustment for sex confirmed a directionally consistent, though borderline, effect (Group: +5.09°, *p* = 0.053; Supplementary Table S5). Among the candidate covariates, only the Pfirrmann grade failed to reach statistical significance (*p* = 0.107) and was excluded from subsequent analyses; all remaining covariates were retained in the residual model.

### 3.2. Reliability and Agreement

Inter-rater ICC values ranged from 0.976 to 1.000 (mean = 0.998; 95% CI [0.995–0.999]). Intra-rater ICC values ranged from 0.977 to 1.000 (mean = 0.998; 95% CI [0.995–0.999]). These results demonstrate highly consistent landmark placement both between and within reviewers.

**Figure 2:**
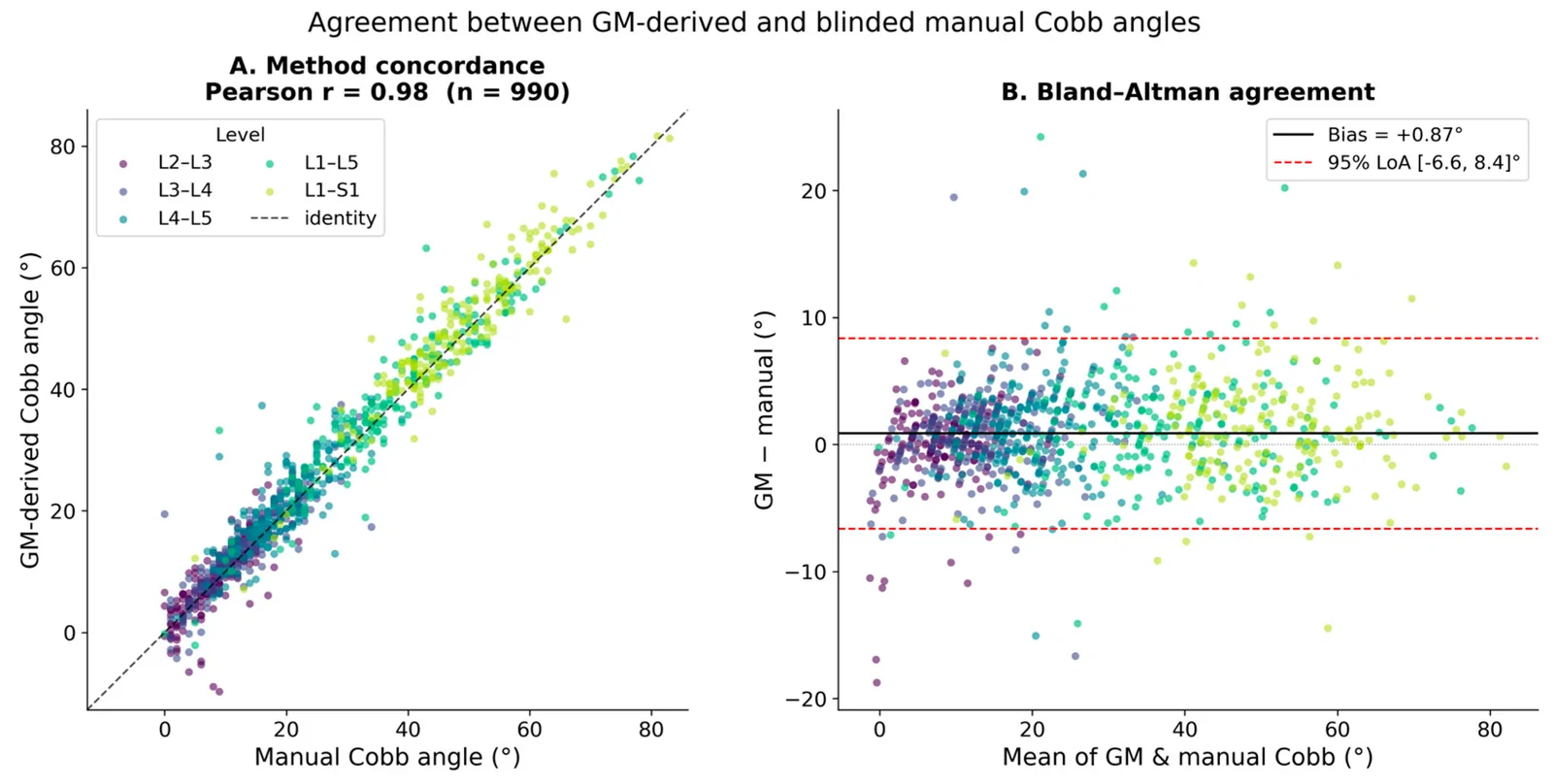
Agreement between GM-derived and manual Cobb angles. Data represent five levels (L2–L3, L3–L4, L4–L5, L1–L5, and L1–S1) across all 198 images (*n* = 990): (**A**) Method concordance (Pearson r = 0.98) with the line of identity. (**B**) Bland–Altman plot showing the mean bias (+0.87°) and 95% limits of agreement [−6.6°, +8.4°]. The L1–L2 and L5–S1 segments are not plotted here; both are reported numerically, alongside all other levels, in Table 2. Colors denote lumbar level.

Correlation was strong at five of seven levels (r = 0.83–0.95), moderate at L5–S1 (r = 0.55, likely reflecting its derivation as a difference of two other angles rather than a direct landmark measurement; see Methods), and weak at L1–L2 (r = 0.16), a near-zero, narrow-range angle at which small absolute disagreement has an outsized relative and correlational impact. Full level-specific results are reported in Table 2; Figure 2 provides a pooled visualization of five selected levels only. Proportional bias was present at L1–L2 and L2–L3 (both *p* < 0.0001) but not elsewhere. Clustering of disagreement within patients was substantial at L1–L2 (ICC = 0.81) and L5–S1 (ICC = 0.52) and negligible at L4–L5 and L1–L5 (ICC ≈ 0).

### 3.3. Postoperative Lumbar Shape Changes Assessed by Geometric Morphometrics

**Figure 3:**
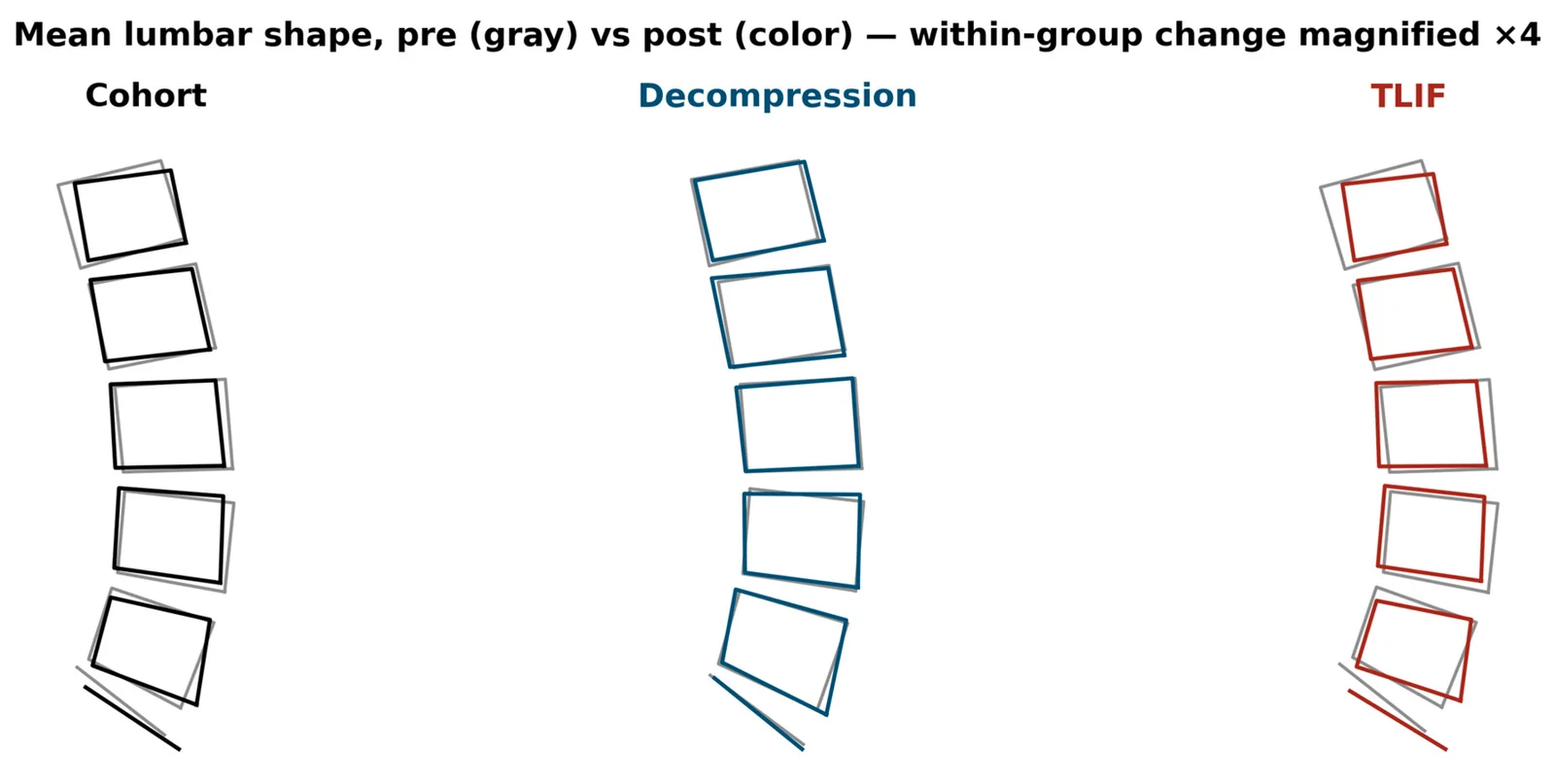
Geometric morphometric shape change after surgery. Mean lumbar wireframes, pre-operative (gray) versus post-operative (color), for the whole cohort, decompression, and TLIF, with the within-group change magnified ×4 for visibility; no significant systematic change was detected after decompression, whereas TLIF altered it, with the largest visually apparent change at the lower lumbar levels (exploratory, uncorrected for multiple comparisons across vertebrae; see Table S4).

**Figure 4:**
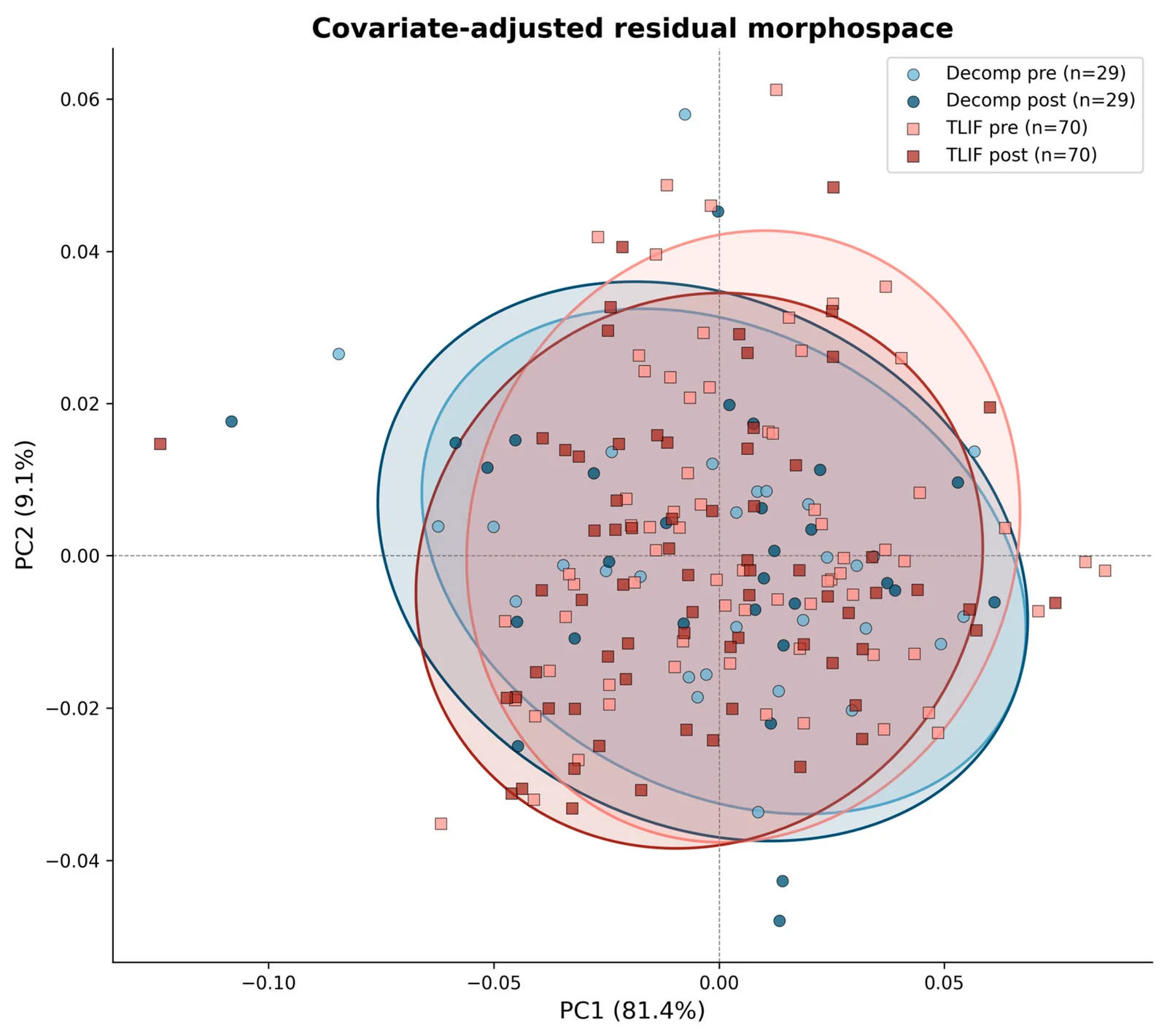
Covariate-adjusted residual morphospace. Principal-component morphospace of covariate-adjusted residual lumbar shape (PC1 81.4%, PC2 9.1%) for all 198 images, by surgical group and timepoint with 95% confidence ellipses. After removal of baseline lordosis, size, and demographic covariates, the four group × timepoint clusters overlap, i.e., no clear separation was visible beyond the lordotic change quantified within patients (Figure 3) and by Cobb analysis.

Within-group analysis showed a significant, systematic shape change after TLIF (sign-flip permutation test on the mean shape-change vector, *p* < 0.001), but not after decompression (*p* = 0.31): TLIF therefore showed a consistent reshaping of the lumbar spine at early postoperative imaging, whereas no significant systematic change was detected after decompression at long-term follow-up (Figure 3). In an unadjusted principal component analysis of the GPA-aligned shapes, the dominant axis (PC1) was descriptively correlated with the L1–S1 angle (r = 0.77, computed across all 198 pre- and post-operative images without accounting for within-patient clustering), and within-patient movement along this axis tracked the change in lordosis (r = 0.58, computed on the 99 independent per-patient change scores). After removing baseline lordosis and the other retained covariates, a separate, covariate-adjusted residual PCA showed a single PC capturing most of the remaining variance (PC1 = 81.4%; PC2 = 9.1%); in this residual morphospace, the surgical groups overlapped (Figure 4).

### 3.4. Postoperative Changes in Lumbar Lordosis Cobb Angles

**Figure 5:**
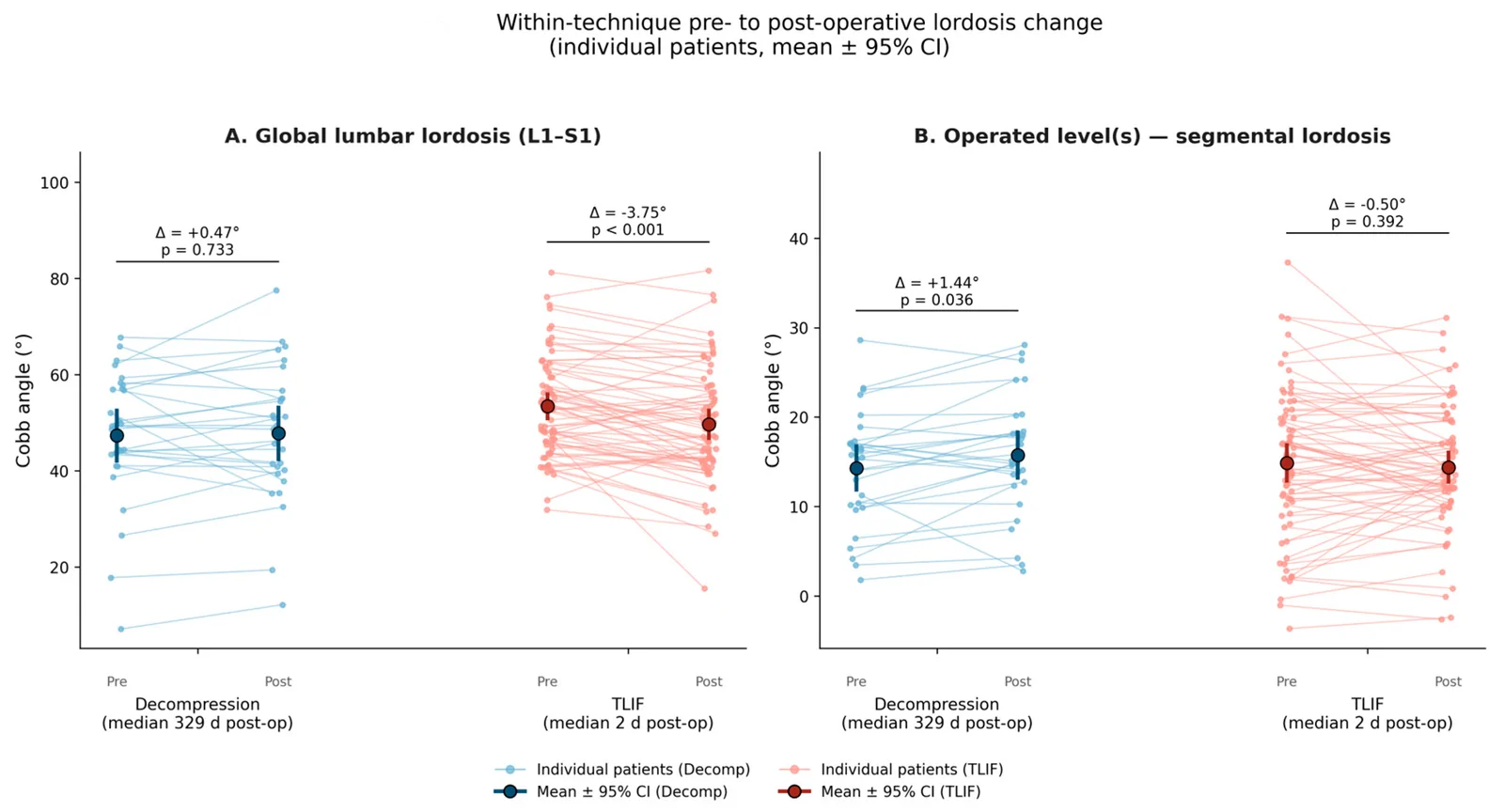
Within-technique pre- to post-operative lordosis change, by surgical group: (**A**) Global lumbar lordosis (L1–S1). (**B**) Operated level(s): mean segmental Cobb angle across each patient’s operated level(s). Light lines and dots show individual patients’ pre-to-post values; bold dots and error bars show the group means ± 95% confidence intervals. Group labels indicate each technique’s median postoperative imaging interval (TLIF: 2 days [IQR 1–52]; decompression: 329 days [IQR 92–588]), reflecting differing routine clinical follow-up schedules for the two techniques rather than a matched comparison timepoint (see Section 2 and Section 4).

Within-group paired *t*-tests showed TLIF patients lost an average of 3.75° ± 7.33° of lordosis at global L1–S1 by early postoperative imaging (median 2 days; *p* < 0.001) (Figure 5A), whereas decompression patients were essentially unchanged by long-term imaging (median 329 days; Δ = +0.47° ± 7.36°, *p* = 0.733) (Figure 5A). At each patient’s operated level(s), decompression showed a small within-group segmental gain (Δ = +1.44° ± 3.52°, *p* = 0.036), while TLIF showed a non-significant change (Δ = −0.50° ± 4.81°, *p* = 0.392) (Figure 5B).

## 4. Discussion

This study applied a combined GM and Cobb angle analysis to a cohort of 99 patients undergoing TLIF or posterior decompression alone, characterizing the within-subject pre- to post-surgical change in lumbar morphology for each technique. Two principal findings emerged. First, TLIF, assessed at early postoperative imaging, showed a significant and systematic change in lumbar shape and a reduction in global lumbar lordosis, while decompression, assessed at long-term imaging, showed a preserved overall configuration and global lordosis, with a small gain in segmental lordosis at the operated level(s). Second, GM-derived measurements showed strong, though level-dependent, correlation with manual Cobb measurements while simultaneously enabling whole-shape analysis that cannot be achieved using conventional Cobb angle assessment. Because the two techniques were imaged at markedly different postoperative intervals as a matter of routine clinical practice, these findings describe each technique’s own postoperative status rather than a validated head-to-head comparison between techniques; together, they support GM as a complementary quantitative imaging approach rather than a replacement for established radiographic measurements.

Contrary to our hypothesis that TLIF would preserve or restore segmental lordosis, TLIF was instead associated with a reduction in global lumbar lordosis at early postoperative imaging (Δ = −3.75°, median interval 2 days). If persistent, this reduction could be clinically relevant, given reported associations between sagittal malalignment, low back pain, and adjacent-segment disease [12,14]. Because these patients were imaged so soon after surgery, this finding reflects an acute postoperative state, potentially including postoperative paraspinal muscle spasm and pain-limited standing extension, rather than a durable structural consequence of the procedure itself [25]. TLIF additionally requires unilateral total facetectomy and posterior element resection for interbody cage placement, a greater degree of local structural disruption than is typical of decompression alone, which provides a plausible anatomical mechanism for acute sagittal-plane change beyond generic perioperative factors [26]. This finding is not necessarily inconsistent with prior literature reporting that TLIF preserves or modestly restores segmental lordosis [15], an effect reported as comparatively small relative to other interbody approaches [17], since those studies generally assess alignment at longer-term follow-up, after fusion consolidation and resolution of acute perioperative effects, rather than in the immediate postoperative period captured here. Whether this acute reduction in lordosis persists, resolves as healing and fusion progress, or reflects a genuine early biomechanical cost of the TLIF approach cannot be determined from a single early postoperative image and would require longer-term follow-up imaging in this group. By contrast, decompression patients, imaged at a median of 329 days, reflecting the later, less routine imaging typical when a formal postoperative film is not obtained immediately after this procedure, showed no significant change in global lordosis (Δ = +0.47°), consistent with prior literature indicating that decompression alone generally preserves or modestly increases lumbar lordosis [18,27]. Decompression additionally showed a small, significant gain in segmental lordosis at the operated level(s) (Δ = +1.44°), which may reflect reduced pain-related posture limitation and improved segmental extension once the compressive pathology was addressed [28,29]. This operated-level gain should be interpreted cautiously, however: it did not reach nominal significance in a sensitivity analysis excluding the subset of patients whose operated level(s) included the less-precise, derived L5–S1 angle (Δ essentially unchanged at +1.47° vs. +1.44°, but *n* = 17, *p* = 0.107), suggesting the full-sample result may be underpowered, though a genuine difference in effect among L5–S1-involved patients, or added measurement noise from the derived L5–S1 angle, cannot be excluded. These proposed mechanisms were not directly measured in the present study and should be regarded as hypotheses for future investigation.

Beyond the technique-specific findings, this study demonstrates the practical value of GM as a complementary quantitative imaging tool. GM-derived Cobb angles showed strong but level-dependent correlation and agreement with an independent, blinded manual measurement at five of seven lumbar levels (r = 0.83–0.95), with the expected exceptions at the near-zero-range L1–L2 angle and the derived L5–S1 angle, both explained by identifiable causes rather than a general failure of the method. Beyond reproducing conventional angular measurements, GM captured the full landmark configuration and resolved the lordotic axis of within-technique shape change with a specificity that a single angular measurement cannot provide. This illustrates the central premise of the study: GM does not replace Cobb angle measurement but adds a complementary, whole-shape perspective that can localize and characterize postoperative change in ways conventional radiographic metrics cannot. Other quantitative alternatives to manual Cobb measurement have also been proposed, including automated/AI-assisted angle detection [30] and whole-spine 3D biplanar (EOS-based) reconstruction [31]; unlike these, which still ultimately report the same standard angular parameters, GM’s distinguishing contribution is its principal-component-based description of shape variation itself, rather than a faster or more automated version of conventional angle measurement.

Several limitations should be acknowledged. First, the retrospective design, relatively small cohort, and imbalance between the TLIF and decompression groups should be considered when interpreting the results, particularly for secondary analyses and subgroup comparisons. The large proportion of the initial surgical cohort excluded for missing postoperative imaging (76/104 excluded patients) may also introduce selection bias if patients who returned for postoperative imaging differ systematically, for example, in symptom burden, complication rate, or follow-up engagement, from those who did not; this is a constraint on the generalizability of the findings to the full surgical population. However, the shape analyses and the covariate-adjusted between-group Cobb comparisons (Supplementary Material) were adjusted for relevant demographic and radiographic covariates, with inference performed using RRPP-based permutation testing; the primary within-group paired *t*-tests for lordosis change were not covariate-adjusted, as each patient serves as their own control. Second, TLIF and decompression patients were imaged at markedly different intervals after surgery (median 2 vs. 329 days) as a matter of routine clinical practice rather than study protocol; this precludes a valid, causally interpretable comparison between techniques. Third, the groups differed at baseline in both lumbar lordosis (decompression patients had approximately 6° less baseline L1–S1 lordosis than TLIF patients) and sex distribution, reflecting real-world surgical selection and introducing potential confounding. However, both variables were included as covariates; baseline alignment accounted for the largest proportion of shape variance, and the lower baseline lordosis of the decompression group was present within each sex. Because the primary outcomes are within-patient pre-to-post changes, in which sex and other time-invariant traits cancel, sex and baseline differences are unlikely to fully explain the within-patient changes on their own. Detailed clinical variables that could further characterize confounding by surgical indication, including body mass index, a structured indication severity score, extent of facetectomy, interbody cage characteristics, and additional pelvic parameters (pelvic tilt, sagittal vertical axis), were not systematically recorded for this retrospective cohort and could not be incorporated; the associations reported here should accordingly be interpreted as associative rather than causal. Detailed radiographic acquisition parameters (equipment, source-to-image distance, and a formal quantitative rotation-tolerance threshold) were not centrally recorded for this retrospective, multi-year cohort; images were screened qualitatively for adequate positioning and minimal rotation prior to digitization (see Exclusion Criteria). Fourth, imaging was evaluated at a single postoperative timepoint and was not correlated with patient-reported outcomes; therefore, the durability and clinical relevance of the observed lordosis changes remain to be established. Finally, the analysis was restricted to sagittal-plane morphology and did not assess three-dimensional spinal alignment. The generally strong, though level-dependent, correlation between GM-derived and manual Cobb measurements suggests that whole-shape morphometric analysis may have potential to complement conventional radiographic assessment, pending further validation of its level-dependent agreement and correlation with clinical outcomes.

## 5. Conclusions

In conclusion, this study demonstrates geometric morphometrics as a feasible and quantitatively interpretable imaging framework for characterizing postoperative lumbar morphology. Landmark-based GM showed excellent inter- and intra-rater reliability and showed strong but level-dependent correlation and agreement with independent manual Cobb angle measurement, while additionally resolving whole-shape, vertebra-level detail that conventional angular measurement cannot capture. As a proof-of-concept application, we used this framework to characterize the within-subject postoperative shape and lordosis change following TLIF and decompression, each assessed at its own respective postoperative imaging interval; because these intervals differed markedly between groups, the observed technique-specific findings should be regarded as hypothesis-generating rather than as a validated comparison of surgical effect and warrant confirmation in a prospective study with matched follow-up. More broadly, this work supports GM as a complementary quantitative tool alongside Cobb angle measurement for future studies of postoperative spinal alignment.

## Figures and Tables

**Table 1:** Baseline demographic and radiographic characteristics. TLIF, transforaminal lumbar interbody fusion. ^a^ *t*-test; ^b^ Pearson χ^2^ test; ^c^ Mann–Whitney U test.

Variable	TLIF (*n* = 70)	Decompression (*n* = 29)	*p* -Value
Age (years), mean ± SD	58.56 ± 13.33	60.76 ± 14.48	0.485 ^a^
Female, *n* (%)	43 (61.4%)	10 (34.5%)	0.026 ^b^
Degenerative spondylolisthesis, *n* (%)	41 (58.6%)	12 (41.4%)	0.180 ^b^
Pfirrmann grade, median (range)	4 (0–5)	4 (0–5)	0.826 ^c^
Number of operated levels, median (range)	1 (1–4)	2 (1–3)	0.079 ^c^
Baseline L1–S1 Cobb (°), mean ± SD	53.42 ± 10.40	47.33 ± 13.77	0.038 ^a^
Pelvic incidence (°)	60.3 ± 12.7 (*n* = 69)	57.9 ± 9.3 (*n* = 28)	0.31 ^a^

**Table 2:** Level-specific agreement between GM-derived and blinded manual Cobb angles. Pearson r, mean bias ± SD, and 95% limits of agreement (LoAs), together with cluster-bootstrap 95% confidence intervals on the lower and upper LoAs (resampled by patient to respect the paired pre/post image structure), are reported per level (*n* = 198 images each). Proportional bias *p*: *p*-value from linear regression of the GM–manual difference on their mean, testing whether disagreement scales with angle magnitude. ICC: intraclass correlation coefficient from a mixed-effects model with a random patient intercept, quantifying clustering of disagreement within patients (ICC ≈ 0 indicates no detectable patient-level clustering).

Level	r	Bias ± SD (°)	95% LoA (°)	95% CI on Lower LoA	95% CI on Upper LoA	Prop. Bias *p*	ICC (Patient)
L1–L2	0.16	−2.57 ± 6.23	[−14.8, 9.6]	[−18.6, −10.2]	[7.0, 11.6]	<0.0001	0.81
L2–L3	0.83	−0.03 ± 3.47	[−6.8, 6.8]	[−9.0, −4.6]	[5.3, 8.1]	<0.0001	0.40
L3–L4	0.89	0.64 ± 3.21	[−5.7, 6.9]	[−7.2, −4.2]	[5.5, 8.4]	0.266	0.14
L4–L5	0.85	1.42 ± 3.93	[−6.3, 9.1]	[−7.7, −4.9]	[7.5, 10.9]	0.050	0.00
L1–L5	0.95	0.97 ± 4.28	[−7.4, 9.4]	[−9.0, −5.9]	[7.4, 11.4]	0.888	0.00
L1–S1	0.95	1.35 ± 3.97	[−6.4, 9.1]	[−7.5, −5.4]	[7.9, 10.4]	0.693	0.02
L5–S1	0.55	−9.19 ± 5.97	[−20.9, 2.5]	[−22.7, −18.8]	[0.9, 3.9]	0.453	0.52

## Data Availability

The data presented in this study are available upon request from the corresponding author.

## Supplementary Materials

The following supporting information can be downloaded at: https://www.mdpi.com/article/10.3390/diagnostics16193177/s1.
