# Supplementary material for: Quantitative Assessment of Postoperative Lumbar Morphology Using Geometric Morphometrics: Within-Subject Changes Following TLIF and Decompression
Source: Diagnostics (Basel). 2026 Sep 30;16(19):3177. doi: 10.3390/diagnostics16193177 (PMC13664590; doi:10.3390/diagnostics16193177)
Supplement: Supplementary file 1 [file diagnostics-16-03177-s001.zip › diagnostics-4563427-supplementary.pdf]

Supplementary Material

Between-Group Comparisons (Secondary, Exploratory Analyses)

Because TLIF and decompression patients in this cohort were imaged at markedly different postoperative intervals as a matter of routine clinical practice (median 2 days for TLIF, 329 days for decompression; see main text Methods and Results), between-group statistical comparisons cannot be interpreted as evidence of a differential surgical effect independent of imaging timing. They are reported here, in full, for transparency and completeness, but should not be read as validating a head-to-head comparison between techniques. The primary, within-technique findings are reported in the main text.

Methods

Two families of between-group models were fitted, in addition to the primary within-technique analyses reported in the main text. First, each patient's pre-to-post shape-change vector (the difference between post-operative and pre-operative GPA-aligned coordinates) was tested against surgical group, adjusted for the retained covariates, by Procrustes ANOVA (Type II sums of squares, 9999 permutations), applied to the whole lumbar configuration and separately to each vertebra. Second, two covariate-adjusted between-group Cobb models were fitted: (i) global lumbar lordosis (L1–S1) as the per-patient change score (post minus pre), modelled on the retained shape-analysis covariates (baseline L1–S1 angle, log centroid size, sex, age, number of operated levels, and degenerative spondylolisthesis) and surgical group (Type II sums of squares); and (ii) the operated-level outcome, modelled as a function of the same covariate set, excluding number of operated levels (implicit in the outcome), and surgical group. Because these two between-group Cobb models (global L1–S1 and operated-level) were pre-specified as a family, significance for each was evaluated against a Bonferroni-corrected threshold ($\alpha = 0.025$) rather than the standard 0.05.

Results

Shape. The per-patient Procrustes distance between the pre- and post-operative configurations was greater after TLIF than after decompression (0.037 vs. 0.030; Mann–Whitney $p = 0.007$; Figure S1). The covariate-adjusted, per-patient shape-change vector also differed significantly by group ($F = 2.78$, $R^2 = 2.8\%$, $p = 0.020$; robust to a Pfirrmann-grade sensitivity check and to exclusion of a single outlying observation, $p = 0.011$), localized at the single-vertebra level to L5 (nominally $p = 0.034$, uncorrected for the five vertebral tests; L1 $p = 0.677$, L2 $p = 0.455$, L3 $p = 0.129$, L4 $p = 0.234$; see also Table S4).

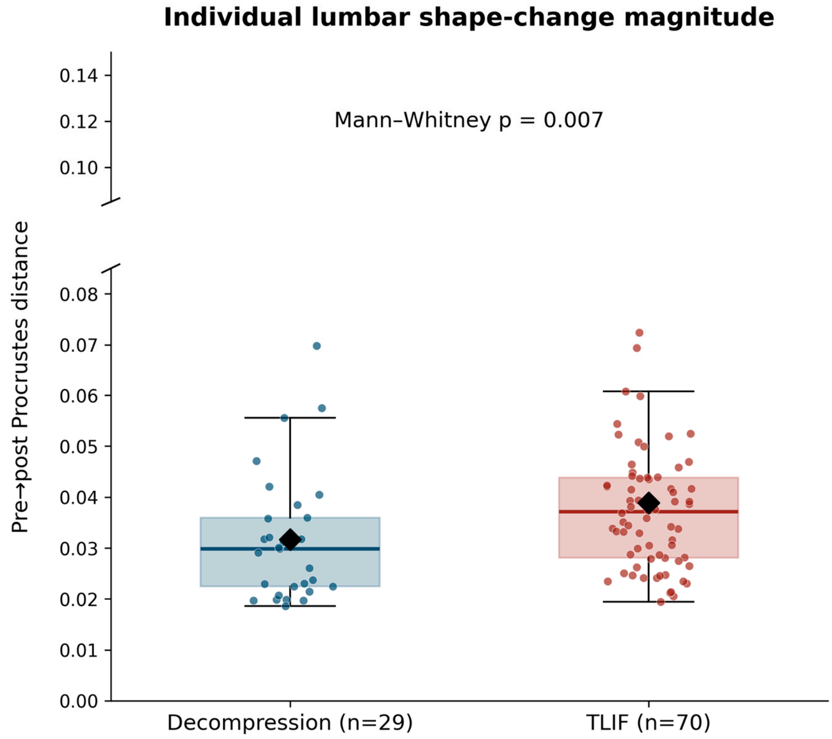

Figure S1. Per-patient Procrustes distance between pre- and post-operative lumbar configurations, by surgical group. TLIF showed a larger individual shape change than decompression (Mann–Whitney p = 0.007). Boxes show median and interquartile range; points show individual patients; diamonds show the group mean. The y-axis is broken to accommodate a single TLIF outlier without compressing the rest of the data.

The per-patient change-score ANCOVA showed a global L1–S1 difference between groups that did not reach the pre-specified Bonferroni-corrected threshold ($F = 4.87$, $p = 0.030$; $\alpha = 0.025$); the difference did cross this threshold when Pfirrmann grade or pelvic incidence was additionally retained in the model ($p = 0.014$). This sensitivity to covariate specification is itself one reason this between-group result is reported as secondary and exploratory rather than confirmatory. No between-group difference was found at the operated level(s) (adjusted surgery effect $p = 0.132$).

Table S1. Number of operated levels by surgical group

Operated levels	TLIF (n = 70)	Decompression (n = 29)
1	48 (68.6%)	14 (48.3%)
2	19 (27.1%)	14 (48.3%)
3	2 (2.9%)	1 (3.4%)
4	1 (1.4%)	0 (0.0%)
Median (range)	1 (1–4)	2 (1–3)

Table S1. Distribution of the number of operated lumbar levels per patient in each group. Groups did not differ significantly (Mann–Whitney U test, p = 0.079).

Table S1b. Exact operated level(s) by surgical group

Operated level(s)	TLIF (n = 70)	Decompression (n = 29)
L1–L2	1	0
L2–L3	0	2
L2–L4	0	1
L2–L5	1	0
L2–S1	1	0
L3–L4	0	2
L3–L5	5	6
L3–S1	1	1
L4–L5	25	6
L4–S1	14	6
L5–S1	22	5

Table S1b. Distribution of exact operated-level combinations by surgical group (preoperative record; totals match Table 1 and Table S1). One patient's source record was coded as "L5‑S2." The original surgical chart was unavailable for verification. Because the landmark configuration extended only to the superior S1 endplate, this case was represented analytically by its measurable L5‑S1 component; no inference was made regarding anatomy caudal to S1.

Table S2. Full Procrustes ANOVA of whole-lumbar shape

Term	df	SS	MS	R^2 (%)	F	Z	p
Baseline L1–S1 angle	1	0.2307	0.2307	24.78	86.99	4.84	0.0001
Log centroid size	1	0.0155	0.0155	1.67	5.85	3.00	0.0007
Sex	1	0.0169	0.0169	1.82	6.38	3.09	0.0001
Age	1	0.0206	0.0206	2.21	7.75	3.46	0.0001
No. operated levels	1	0.0115	0.0115	1.24	4.34	2.56	0.0038
Degenerative spondylolisthesis	1	0.0075	0.0075	0.81	2.84	1.96	0.0265
Pfirrmann grade	1	0.0047	0.0047	0.50	1.77	1.26	0.1074
Group (TLIF vs decompression)	1	0.0036	0.0036	0.39	1.37	0.85	0.2027
Timepoint (pre vs post)	1	0.0064	0.0064	0.69	2.41	1.77	0.0402
Group × Timepoint	1	0.0015	0.0015	0.17	0.58	−0.65	0.7387
Residuals	187	0.4959	0.0027	53.27			
Total	197	0.9309		100.0			

Table S2. Full Procrustes ANOVA (Type II sums of squares; residual randomization, 9999 permutations) on GPA-aligned whole-lumbar coordinates (48 landmarks, 198 specimens). Pfirrmann grade was the only candidate covariate not retained. Surgery (Timepoint) significantly altered overall shape, but the two techniques did not differ on this pooled test (Group × Timepoint, p = 0.74). Because this pooled screening model did not account for within-patient dependence, its Group, Timepoint, and Group × Timepoint terms were not used for inference; surgical-effect inference was based on the repeated-measures-safe within-patient change-vector analyses reported in the main text and Supplementary Table S4.

Table S3. Proportion of lumbar shape variance explained by each covariate

Covariate	R^2 (% of total shape variance)	Retained in adjusted model
Baseline L1–S1 angle	24.8	Yes
Age	2.2	Yes
Sex	1.8	Yes
Log centroid size	1.7	Yes
No. operated levels	1.2	Yes
Degenerative spondylolisthesis	0.8	Yes
Pfirrmann grade	0.5	No (p = 0.107)
Retained covariates (combined)	≈32.5	—

Table S3. Variance in whole-lumbar shape explained by each candidate covariate (from Table S2). Baseline sagittal alignment (L1–S1) was by far the dominant source of shape variation. The six retained covariates jointly explained ~32.5% of total shape variance and were removed before testing the surgical effect.

Sensitivity analysis: forcing Pfirrmann grade into both the primary shape-change model and the L1–S1 change-score model, despite its non-significance in the covariate-screening model (p = 0.107), did not materially change the surgical-group effect (shape change: p = 0.021 vs. 0.020; L1–S1 change: p = 0.030 vs. 0.030), supporting the covariate-selection strategy used in the main analysis.

Sensitivity analysis: excluding the single outlying observation in Figure S1 did not change the conclusions: the Mann–Whitney comparison remained significant (p = 0.009, n = 98), as did the covariate-adjusted, repeated-measures shape-change test (p = 0.011, n = 98).

Sensitivity analysis: excluding the 50 patients whose operated level(s) included the less-precise, derived L5–S1 angle left the direction of both findings, and the magnitude of the decompression finding, similar (TLIF: Δ = −1.74° vs. −0.50° in the full sample; decompression: Δ = +1.47° vs. +1.44°), but the decompression result no longer reached

nominal significance in this smaller subsample (n = 17, p = 0.107, vs. p = 0.036 in the full sample; TLIF n = 32, p = 0.089 vs. p = 0.392), consistent with reduced statistical power at the smaller sample size, though a genuine difference in the underlying effect among patients with L5–S1 involvement, or added noise from the less-precise derived L5–S1 measurement itself, cannot be excluded.

Table S4. Per-vertebra Procrustes ANOVA (repeated-measures-safe, per-patient shape-change vector)

Vertebra	Group effect p
L1	0.677
L2	0.455
L3	0.129
L4	0.234
**L5**	**0.034**

Table S4. Single-vertebra, repeated-measures-safe Procrustes tests (each vertebra analysed separately using the per-patient shape-change vector, adjusted for the retained covariates). L5 showed a nominally significant group effect on shape change (p = 0.034, highlighted); no other vertebra reached nominal significance. No correction for multiple comparisons across the five vertebrae was applied; this result should accordingly be treated as exploratory.

Table S5. Baseline characteristics stratified by sex

Variable	Female (n = 53)	Male (n = 46)	p-value
Age (years), mean ± SD	61.5 ± 13.9	56.6 ± 13.0	0.072
Degenerative spondylolisthesis, n (%)	36 (68%)	17 (37%)	0.003
Pfirrmann grade, median	4	3	0.042
Number of operated levels, median	1	1	0.859
Baseline L1–S1 Cobb (°), mean ± SD	53.9 ± 12.2	49.0 ± 10.8	0.037
TLIF, n (%)	43 (81%)	27 (59%)	0.026

Table S5. Baseline characteristics by sex. Women had greater lumbar lordosis and a higher prevalence of degenerative spondylolisthesis. The lower baseline L1–S1 lordosis of the decompression group was similar in magnitude within each sex (women: decompression 48.0° vs. TLIF 55.3°; men: decompression 47.0° vs. TLIF 50.5°). A linear model of baseline L1–S1 angle on Group adjusted for Sex confirmed a directionally consistent effect after formal adjustment (Group: +5.09°, p = 0.053), suggesting the group difference is not primarily driven by sex, though this adjusted estimate was borderline and did not reach conventional

significance. Continuous variables, independent-samples t-test; ordinal variables, Mann–Whitney U test; categorical variables, Pearson χ^2 test.
